# Assessing the Relationship Between Depressive Symptoms and Menopausal Quality of Life Among Academic Women in Saudi Arabia

**DOI:** 10.3390/healthcare13131557

**Published:** 2025-06-30

**Authors:** Sally Mohammed Farghaly Abdelaliem, Noha Mohamed Mahmoud Hassan, Aljory Alqahtani, Lama Alamer, Noura Alhomaid, Hessa Alsubaie, Rania Alsaeed, Dalal Al-Qahtani, Mudhawi Alenazi

**Affiliations:** 1 Nursing Management and Education Department, College of Nursing, Princess Nourah bint Abdulrahman University, P.O. Box 84428, Riyadh 11671, Saudi Arabia; 2 Maternity and Pediatric Nursing Department, College of Nursing, Princess Nourah bint Abdulrahman University, P.O. Box 84428, Riyadh 11671, Saudi Arabia; nmhassan@pnu.edu.sa; 3 College of Nursing, Princess Nourah bint Abdulrahman University, P.O. Box 84428, Riyadh 11671, Saudi Arabia; 443005714@pnu.edu.sa (A.A.); 443005699@pnu.edu.sa (L.A.); 443005637@pnu.edu.sa (N.A.); 443005688@pnu.edu.sa (H.A.); 443005542@pnu.edu.sa (R.A.); 443005396@pnu.edu.sa (D.A.-Q.); 443005638@pnu.edu.sa (M.A.)

**Keywords:** menopause, quality of life, depression, anxiety, stress, mental health, menopausal symptoms, psychological distress, women’s health, Saudi Arabia

## Abstract

**Background/Objective**: Menopause marks a significant life transition for women, signaling the end of reproductive ability and triggering various physiological and psychological changes. During this phase, women may experience a range of physical and emotional challenges that can affect their quality of life. This study aims to assess the relationship between women’s mental health and their menopausal quality of life. **Methods**: A descriptive correlational study was conducted among 350 women aged 45–60 years who had either experienced menopausal symptoms or undergone menopause. Using convenience sampling, participants were recruited from academic institutions in Riyadh, Saudi Arabia. Data were collected using validated tools, including the Menopause Rating Scale (MRS) and the Depression Anxiety Stress Scales (DASS-21). Descriptive and inferential statistical analyses assessed symptom severity and its association with mental health and quality of life. **Results**: Findings indicated that 27.8% of participants experienced moderate to severe menopausal symptoms. Urogenital symptoms were the most common, reported by 59% of women. Significant correlations were observed between menopausal symptoms and high levels of depression (63%), anxiety (60%), and stress (58%), all of which significantly impacted quality of life. **Conclusions**: Menopausal symptoms have a profound impact on both physical and mental health, significantly affecting quality of life. Depression, in particular, was found to be the most influential factor. These findings highlight the need for integrated healthcare approaches that address both physical and psychological aspects of menopause.

## 1. Introduction

Menopause represents a significant biological milestone in a woman’s life, signifying the permanent cessation of menstruation following the decline of ovarian follicular activity [1]. Globally, the average age of natural menopause ranges between 45 and 55 years [2]. The menopausal transition, or perimenopause, usually begins 5 to 8 years prior to the final menstrual period and is characterized by fluctuating hormonal levels, particularly estrogen and progesterone, which contribute to a wide spectrum of somatic, vasomotor, and psychological symptoms [3].

Among the numerous challenges encountered during menopause, mental health disturbances—particularly depressive symptoms—are increasingly recognized as significant concerns. Studies have consistently demonstrated that women in the menopausal transition are at elevated risk for developing depressive symptoms compared to premenopausal or postmenopausal counterparts [3,4]. For example, findings from the Study of Women’s Health Across the Nation (SWAN) indicated that approximately 23% of midlife women experience moderate to severe depressive symptoms during the menopausal transition, compared to 15% in premenopausal stages [5]. These depressive symptoms may be influenced by both hormonal fluctuations (particularly declining estrogen levels that affect serotonin and other neurotransmitters) and psychosocial stressors such as caregiving burdens, evolving family dynamics, financial pressures, and changing self-identity [3,6].

The presence of depressive symptoms during menopause exerts a profound negative impact on women’s quality of life (QoL), a multidimensional construct encompassing physical, psychological, social, and sexual well-being [7,8]. Several studies have shown that greater severity of depressive symptoms is directly associated with poorer QoL scores across these domains. In a large cross-sectional study, Avis et al. reported that women with higher depressive symptom scores exhibited significantly diminished QoL related to energy/fatigue, role limitations, emotional well-being, and social functioning [6]. Moreover, sleep disturbances, vasomotor symptoms (such as hot flashes and night sweats), and sexual dysfunction often co-occur with depressive symptoms, further compounding the overall burden on QoL [9].

Importantly, the experience of menopause, including its psychological and QoL outcomes, does not occur in isolation but is heavily shaped by broader sociocultural, economic, and individual contexts [10]. For example, cultural attitudes toward aging and menopause can either exacerbate or mitigate psychological distress. In some cultures, menopause is perceived as a natural and respected transition, whereas in others it may carry negative connotations of aging and loss of femininity, contributing to greater psychological morbidity [11,12]. Social support networks, healthcare access, educational level, and socioeconomic status further influence how women experience and manage menopausal challenges [13].

Therefore, examining the intricate relationship between depressive symptoms and QoL during menopause is of critical importance. Such an understanding can inform the development of targeted, culturally sensitive interventions that promote mental well-being, mitigate depressive symptomatology, and enhance QoL outcomes during this pivotal life stage. Moreover, addressing these issues holds important public health significance, as improving mental health and QoL among menopausal women can contribute to broader societal benefits, including reduced healthcare costs, improved workforce participation, and enhanced familial stability [12,13].

Study Aim: The study aims to assess the relationship between depressive symptoms and menopausal quality of life among women.

Research Questions: What is the relationship between depressive symptoms and quality of life among women undergoing menopause, specifically regarding mood, anxiety, cognitive function, and overall well-being?

Hypothesis: There is a significant positive correlation between depressive symptoms and menopausal quality of life, such that lower levels of depressive symptoms are associated with higher menopausal quality of life among women.

## 2. Materials and Methods

Research Design: This study employed a quantitative, non-experimental, correlational, cross-sectional research design.

Research Setting: This study was conducted at an academic institution in the Kingdom of Saudi Arabia. The sample consisted of women employed at the university, including faculty members, administrative staff, and support personnel. Recruiting participants from this academic setting allowed us to explore menopausal quality of life and mental health symptoms, specifically depressive symptoms, among a relatively homogenous professional group who may share unique occupational demands, work-related stressors, and access to institutional healthcare resources. Such factors may influence both the severity and reporting of menopausal symptoms and psychological well-being. Moreover, academic women may experience distinctive stress patterns related to balancing professional responsibilities, family roles, and sociocultural expectations specific to the Saudi Arabian context. While this sample provided valuable insight into the menopausal experiences of women in academic employment, we recognize that the findings may not fully represent the broader female population in Saudi Arabia, including women from other occupational sectors or unemployed women. Differences in sociodemographic profiles, work environments, healthcare access, and cultural experiences across various groups may influence menopausal experiences and mental health outcomes. These considerations have been acknowledged in the study limitations.

Sampling: The study population consisted of approximately 2000 women aged 45 to 60 years who were employed at an academic institution in Riyadh, Saudi Arabia. From this population, a sample of 350 women was recruited to participate in the study. The sample size was determined using Epi Info software version 7.2, based on a 95% confidence level, a 5% margin of error, and an assumed prevalence rate appropriate for the target population. Inclusion criteria were as follows:

- Women in perimenopause (irregular menstruation with menopausal symptoms) or postmenopause (≥12 months of amenorrhea without other causes).
- Women still menstruating but reporting clinically significant menopausal symptoms (defined as a total score ≥17 on the Menopause Rating Scale [MRS] [14], based on established cutoffs for moderate-to-severe symptom burden).
- No pre-existing diagnosed mental health disorders (self-reported or documented).

Exclusion criteria included the following:

- Women with known psychiatric conditions (e.g., depression, anxiety disorders).
- Women outside the age range (45–60 years).
- Those unable or unwilling to provide informed consent.

Sampling techniques: A non-probability technique; convenience sample participants are selected based on their ease of access or availability to the researcher.

Instruments: In this study, three valid Arabic versions of instruments were used to assess the study variables.

### 2.1. Tool (1): Menopause-Specific Quality of Life Questionnaire (MENQOL)

The Menopause-Specific Quality of Life Questionnaire (MENQOL), developed by Hilditch et al. (1996) [15], was used to assess the impact of menopausal symptoms on quality of life. The MENQOL includes 29 items divided into four domains: vasomotor (3 items), psychosocial (7 items), physical (16 items), and sexual (3 items). Each item is rated on a 6-point Likert scale ranging from 0 (“not bothered”) to 5 (“extremely bothered”), reflecting symptom bother over the past month. Subscale scores for each domain were calculated by summing the item scores and dividing by the number of items in that domain to obtain a mean score, while the total MENQOL score was obtained by averaging all items. Higher scores indicate greater symptom burden and lower quality of life. Since MENQOL does not have established clinical cut-off scores, we categorized quality of life impact based on sample percentiles as follows: good (≤25th percentile), fair (>25th to ≤75th percentile), and poor (>75th percentile).

### 2.2. Tool (2): Depression Anxiety Stress Scale (DASS-21)

The Depression Anxiety Stress Scales (DASS-21), developed by Lovibond and Lovibond (1995) [16], was used to assess psychological distress across three subscales, including depression, anxiety, and stress, with seven items per subscale. Participants rated each item based on their experiences over the past week using a 4-point Likert scale ranging from 0 (“never”) to 3 (“almost always”). Subscale scores were calculated by summing the item scores within each domain and multiplying by 2 to match the original scoring system of the DASS-42, resulting in a score range of 0 to 42 for each subscale. Severity was classified according to established cut-offs: for depression, normal (0–9), mild (10–13), moderate (14–20), severe (21–27), and extremely severe (≥28); for anxiety, normal (0–7), mild (8–9), moderate (10–14), severe (15–19), and extremely severe (≥20); and for stress, normal (0–14), mild (15–18), moderate (19–25), severe (26–33), and extremely severe (≥34).

### 2.3. Tool (3): Menopause Rating Scale (MRS)

The Menopause Rating Scale (MRS), developed by Heinemann et al. (2004) [14], was used to evaluate menopausal symptom severity across three domains: somatic (4 items), psychological (4 items), and urogenital (3 items). Each item is rated on a 5-point Likert scale ranging from 0 (“none”) to 4 (“very severe”), assessing current symptoms. Subscale scores were calculated by summing the item scores for each domain, yielding possible ranges of 0–16 for somatic and psychological domains and 0–12 for the urogenital domain. The total MRS score was calculated by summing all 11 items, resulting in a total score range of 0–44. Severity was categorized as mild (total 1–16), moderate (17–23), and severe (≥24). The Arabic validated version of the MRS was used in this study.

#### 2.3.1. Validity and Reliability

The three tools were adjusted, then translated into Arabic and back into English. The tools were then submitted to a panel of five experts (three professors from the Maternity and Pediatric Department and two lecturers from the psychiatric nursing department) who examined and assessed the content validity and offered feedback on the content, question types, and item clarity.

#### 2.3.2. Psychometric Evaluation and Pilot Testing

As the questionnaires were used in the Arabic language, a rigorous forward-backward translation procedure was applied for all instruments (MENQOL, DASS-21, and MRS) to ensure linguistic and conceptual equivalence. The process involved independent bilingual experts performing forward translation into Arabic, followed by back-translation into English by separate bilingual translators. Discrepancies were discussed and resolved through expert panel review to ensure cultural relevance and content validity for the Saudi population.

Following translation, a pilot study was conducted on 10% of the target population (n = 35) to assess item clarity, cultural appropriateness, and overall feasibility of the survey instruments. Feedback from pilot participants was used to refine wording and ensure participant understanding. These pilot participants were not included in the final study sample.

The psychometric properties of the Arabic versions of the instruments were subsequently evaluated. Construct validity was assessed using exploratory factor analysis (EFA) with principal component analysis and varimax rotation. The EFA results supported the multidimensional structures of all three scales. For the MENQOL, the four-factor solution corresponded to vasomotor, psychosocial, physical, and sexual domains, explaining a substantial proportion of total variance. Similarly, the MRS showed three distinct domains—somatic, psychological, and urogenital—consistent with the original instrument structure. For the DASS-21, the factor structure yielded three factors corresponding to depression, anxiety, and stress, confirming the instrument’s multidimensional design [15,16].

Internal consistency reliability was assessed using Cronbach’s alpha for each subscale. The MENQOL subscales yielded Cronbach’s alpha values ranging from 0.84 to 0.92, indicating high internal consistency. The MRS subscales demonstrated Cronbach’s alpha values between 0.81 and 0.89, while the DASS-21 subscales showed alpha values of 0.86 for depression, 0.84 for anxiety, and 0.88 for stress, reflecting strong internal consistency for all domains. These results confirm the reliability and construct validity of the translated instruments for use in the current study population.

Data Gathering Procedure: A questionnaire was applied to a group of women undergoing menopause from January 2025 to the end of April 2025 at an academic institution. The process involved designing practical questions, selecting appropriate respondents, and analyzing the gathered data to draw meaningful insights. The survey took approximately 15 to 30 min to complete.

Statistical Analysis: Data were analyzed using IBM SPSS Statistics (Version 26) and the PROCESS macro for mediation modeling (Hayes, 2018) [17]. Descriptive statistics (means, standard deviations, frequencies, and percentages) summarized demographic and clinical variables. Prior to inferential analyses, normality assumptions were verified using Shapiro–Wilk tests *p* > 0.05 for all continuous variables) and visual inspection of Q-Q plots, confirming that Menopause Rating Scale (MRS), Depression Anxiety Stress Scales (DASS-21), and Menopause-Specific Quality of Life (MENQOL) scores followed normal distributions. This justified the use of parametric tests, including Pearson’s correlation coefficients (r) to evaluate linear relationships between variables. Correlation strengths were interpreted using Cohen’s (1988) criteria: coefficients of 0.10–0.29, 0.30–0.49, and ≥0.50 indicated weak, moderate, and strong relationships, respectively [18]. To examine the mediating role of menopausal symptoms (MRS) in the relationship between mental health (DASS-21) and quality of life (MENQOL), we conducted mediation analysis via Hayes’ PROCESS Model 4 with 5000 bootstrap samples. The Sobel test confirmed the significance of indirect effects. The proportion of mediation was calculated as [(indirect effect/total effect) × 100], where total effects represented the sum of direct and indirect pathways (Preacher & Kelley, 2011) [19]. All statistical tests were two-tailed, with *p* ≤ 0.05 considered significant.

Ethical Consideration: The Institutional Review Board of a governmental university at Riyadh (N: 25-0051) excused the study from ethical assessment. The subjects provided informed consent after being told about the study’s goal. Confidentiality and anonymity were ensured by assigning a code number to each questionnaire. The students were assured that their information would be kept strictly confidential and used only for research purposes. The ability to exit the study at any time was ensured.

## 3. Results

Table 1 presents the socio-demographic characteristics of the study participants (n = 350). The sample included both postmenopausal women (≥12 months amenorrhea) and perimenopausal women (experiencing menopausal symptoms with irregular menstruation). Of the total sample, 228 women (65.1%) were postmenopausal, while 122 (34.9%) were perimenopausal. The mean age was 53.04 ± 7.11 years, with most participants (54.9%) aged 51–55 years. Regarding education, 52.3% held university degrees. The majority were married (77.4%).

Table 2 presents a mean score of 3.57 ± 2.26 for somatic symptoms. Among women reporting mild symptoms, sleep problems (50.0%) and hot flashes (57.7%) were most frequently reported. Moderate to severe somatic symptoms affected 31.7% to 32.2% of the total sample. Psychological symptoms showed a mean score of 3.55 ± 2.6, with anxiety (66.6%) and depressive mood (58.0%) being most prevalent in the mild symptom group, while moderate to severe symptoms affected 32.3% to 15.4% of participants overall. Urogenital symptoms demonstrated the highest mean score (6.17 ± 3.82), with physical/mental exhaustion (66.6%) and sexual problems (53.4%) most common among mild cases, while moderate to severe urogenital symptoms were reported by 18.9% to 10.3% of the total sample. The overall MRS mean score was 13.29 ± 7.44, with 40.3% experiencing moderate and 31.4% severe symptoms across all domains.

Table 3 presents the mean and standard deviation (SD) scores for the Menopause-Specific Quality of Life Questionnaire (MENQOL) among 350 participants. Overall, the MENQOL score was 67.46 ± 36.85, indicating that 27.8% of participants experienced moderate to severe quality of life impairment due to menopausal symptoms.

Table 4 shows the Pearson correlation coefficients examining relationships between menopausal symptom severity (MRS), mental health (DASS-21), and quality of life (MENQOL). The analysis reveals several key findings. First, a significant moderate correlation exists between overall menopausal symptoms and mental health (r = 0.346, *p* < 0.001), indicating that women experiencing more severe menopausal symptoms tend to report poorer mental health. Second, quality of life shows significant but weaker association with menopausal symptoms (r = 0.227, *p* < 0.001) compared to its stronger relationship with mental health (r = 0.635, *p* < 0.001). This pattern suggests that while menopausal symptoms contribute to diminished quality of life, mental health factors may play a more substantial role.

**Figure 1:**
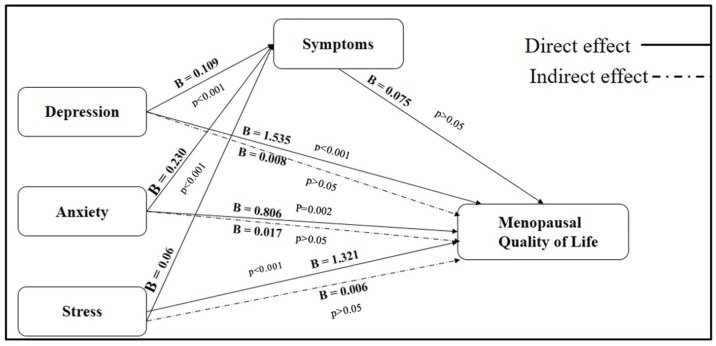
Mediating role of the Menopause Rating Scale (MRS) in the relationship between DASS-21 and Menopause-Specific Quality of Life (MENQOL) (n = 350).

The findings, presented in Table 5 and Figure 1, demonstrate that menopausal symptoms partially mediate the effects of psychological distress on quality of life outcomes. Regarding depression, the total effect on MENQOL was statistically significant (B = 1.543, *p* < 0.001). A large portion of this effect was attributable to a direct pathway (B = 1.535, *p* < 0.001), representing 34.1% of the total relationship. The indirect effect through menopausal symptoms was relatively modest (B = 0.008), contributing 25.8% of the total effect, indicating partial mediation. Similarly, anxiety was found to have a significant total effect on MENQOL (B = 0.823, *p* < 0.001). The direct effect (B = 0.806, *p* = 0.002) accounted for 34.5% of the total impact, while the indirect effect via menopausal symptoms (B = 0.017) explained 26.0% of the relationship. These results suggest that anxiety also exerts both direct and partially mediated influences on quality of life. In the case of stress, the total effect on MENQOL was significant (B = 1.327, *p* < 0.001), with the direct effect contributing 40.5% (B = 1.321, *p* < 0.001). The indirect effect through menopausal symptoms was modest (B = 0.006) but accounted for 38.1% of the total relationship, suggesting a substantial partial mediation effect. These pathways are visually illustrated in Figure 1, where solid lines represent direct effects and dashed lines denote indirect effects mediated through menopausal symptoms. Among the psychological predictors, depression had the strongest direct influence on MENQOL (B = 1.535), followed by stress (B = 1.321) and anxiety (B = 0.806). All three psychological factors were significantly associated with menopausal symptoms, with the strongest association found for depression (B = 0.109), followed by anxiety (B = 0.230) and stress (B = 0.086). Interestingly, while the pathway from menopausal symptoms to MENQOL was positive (B = 0.075), it did not reach statistical significance (*p* = 0.730). This suggests that although menopausal symptoms play a mediating role, their direct impact on quality of life may be less influential compared to the strong direct effects exerted by psychological.

## 4. Discussion

The present study found that 54.9% of participants were within the 51–55 age range, aligning with the typical onset period of menopause. This distribution reflects global demographic patterns where the menopausal transition is most pronounced in the early 50s, a time characterized by heightened symptom severity due to progressive ovarian failure and hormonal fluctuation. Supporting this, Santoro et al. (2021) [20] emphasized that women in their early 50s frequently exhibit intensified vasomotor, psychological, and urogenital symptoms, consistent with the natural course of reproductive aging. Analysis of menopausal symptom domains showed that urogenital symptoms were the most prominent, followed by psychological and somatic complaints. This pattern is physiologically justified by the decline in estrogen levels, which significantly impacts the urogenital tract, leading to vaginal dryness, sexual dysfunction, and urinary incontinence. These findings correspond with studies by Harlow et al. (2020) [21], who found that genitourinary syndrome of menopause (GSM) was the most persistent and distressing symptom cluster among postmenopausal women. Moreover, psychological symptoms such as anxiety and mood disturbances were prevalent, potentially linked to estrogen’s role in serotonin regulation, which modulates emotional states (Schmidt et al., 2020) [4].

The severity of somatic symptoms, including hot flashes and sleep disturbances, can be attributed to autonomic instability caused by hormonal shifts, affecting thermoregulation and circadian rhythms. Avis et al. (2019) [22] supported this explanation, reporting high levels of vasomotor and sleep-related complaints in midlife women. However, contrasting results were observed in a study by Greendale et al. (2021) [23], which identified somatic symptoms as more prominent than urogenital symptoms in certain ethnic groups, suggesting cultural, genetic, and reporting differences as influencing factors. The psychological burden among participants was particularly notable, with high levels of depression, anxiety, and stress among those experiencing severe menopausal symptoms. The impact of estrogen withdrawal on the hypothalamic-pituitary–adrenal (HPA) axis likely contributes to these emotional disturbances. These findings resonate with those of Soares et al. (2020) [24], who demonstrated that fluctuations in estrogen levels can destabilize mood through neuroendocrine mechanisms, significantly impairing emotional regulation.

The symptom distribution across the physical, psychosocial, sexual, and vasomotor domains confirmed that physical symptoms were the most frequently reported and impactful, particularly fatigue, muscle pain, and sleep disturbances. Psychosocial symptoms like poor memory, sadness, and anxiety were also prevalent. Although sexual symptoms were less frequently reported, they still posed significant implications for relational and emotional well-being. Vasomotor symptoms were the least reported, possibly due to individual, environmental, or cultural factors influencing symptom perception or reporting. These trends are consistent with findings by Johnson L et al. (2020) [25], who observed that physical symptoms—especially fatigue and sleep issues—are among the most disabling during menopause. However, not all studies agree. Hunter and Mann (2010) [26] argued that emotional distress during menopause might often result more from environmental stressors or existing mental health conditions than menopause itself. This reinforces the need for personalized, multifactorial care models that address physical, emotional, and contextual variables.

Correlational analysis further demonstrated significant associations between menopausal symptoms and psychological distress, with physical symptoms being strongly linked to reduced quality of life. These results suggest that menopausal women, especially in midlife, experience compounded challenges that affect both physical and emotional well-being. This supports the findings of Dennerstein et al. (2019) [27], who highlighted that emotional vulnerability can amplify the perception of menopausal symptoms. Furthermore, Freeman et al. (2021) [28] affirmed that menopausal symptoms significantly impact women’s quality of life, particularly through the physical and psychological domains. However, some scholars argue that these relationships are not exclusively hormonal. For instance, Guerrero-González et al. (2021) [29] noted that psychological outcomes in menopause may also be shaped by external stressors, coping mechanisms, and social support rather than solely physiological changes. These findings underscore the importance of adopting a biopsychosocial model when evaluating menopausal experiences.

Overall, this study demonstrates that depression, anxiety, and stress significantly influence menopause-related quality of life, with the Menopause Rating Scale (MRS) serving as a valuable mediator in assessing the overall burden. Depression was found to have a particularly dominant effect, which aligns with the findings of Lee, S. et al. (2021) [30] that emphasized its pervasive impact on cognition and symptom interpretation. Unlike stress and anxiety, which are often intensified by physical symptoms, depression may exert a more direct influence through its effect on mental resilience and coping.

The findings indicate that psychological distress (depression, anxiety, and stress) significantly impacts quality of life during menopause, both directly and indirectly through menopausal symptoms. The partial mediation by menopausal symptoms highlights the complex interplay between psychological factors and physical symptoms in determining quality of life outcomes. This could be attributed to the fact that menopause involves major hormonal shifts, especially a decline in estrogen levels during menopause, which can influence neurotransmitters like serotonin and GABA. This contributes to mood disorders, making women more vulnerable to psychological issues like depression, anxiety, and stress. The relationship between hormonal changes and psychological elements can intensify menopausal symptoms, thus affecting overall quality of life. This is in line with Lee, S. et al. [30] who reported a direct effect of low menopausal quality of life on ageing anxiety and a mediating effect of low depression and higher life satisfaction on ageing anxiety. Moreover, Mosconi et al. (2021) [31] highlights how changes in neuroendocrine and neurotransmitter systems, related to decreased estrogen, negatively affect the psycho-physical well-being of women, potentially explaining the increased incidence of mental disorders in the postmenopausal era.

### Strengths and Limitations

This study presents several noteworthy strengths. Primarily, it offers a comprehensive examination of the relationship between women’s mental health and their menopausal quality of life, an area that remains underexplored, particularly within the regional context. The inclusion of validated instruments—namely, the Menopause Rating Scale (MRS) and the Depression Anxiety Stress Scales (DASS-21)—enhances the credibility and reproducibility of the findings. The relatively large sample size of 350 participants from an academic institution contributed to the statistical power and the identification of significant associations across multiple domains of menopausal symptoms and psychological distress. While the sample was specific to working women in an academic setting, it included diversity in age (45–60 years), menopausal status (65.1% postmenopausal, 34.9% perimenopausal), and educational background (14.3% to 52.3% university educated). However, several limitations should be acknowledged. First, recruiting participants exclusively from an academic institution may introduce a sampling bias, as academic women may experience unique occupational stressors, better healthcare access, and higher educational levels that are not representative of the general female population in Saudi Arabia. This may limit the generalizability of the findings to other professional groups, unemployed women, or women from different socioeconomic backgrounds. Second, as with all self-reported data, the potential for recall bias and social desirability bias exists, particularly in sensitive areas like sexual health and emotional well-being. Third, the cross-sectional design precludes any causal interpretations regarding the relationship between depressive symptoms and menopausal quality of life. Finally, the absence of clinical measures, such as hormonal assays, limits the ability to directly link reported symptoms with physiological changes, thus necessitating cautious interpretation of the findings. Recognizing menopause as a critical life stage that can affect mental well-being and quality of life is essential for informing clinical practice, health education, and policy planning.

## 5. Conclusions

The findings of this study highlight the intricate and significant relationship between menopausal symptoms and women’s mental health. Depression, anxiety, and stress were shown to adversely affect various dimensions of menopausal quality of life, with depression emerging as the most impactful. The predominance of urogenital symptoms, followed by psychological and somatic complaints, reflects the multifaceted burden of menopause on women’s daily functioning and emotional well-being. These symptoms appear to be influenced not only by hormonal changes but also by sociodemographic factors such as age, education, and marital status. Women with higher education levels and stronger social support—especially from a spouse—were more likely to report fewer or less severe symptoms, suggesting a protective role of psychosocial resources. Overall, the results underscore the necessity of adopting a holistic perspective on menopausal health—one that goes beyond physiological symptoms to encompass emotional, cognitive, and social dimensions. Recognizing menopause as a critical life stage that can affect mental well-being and quality of life is essential for informing clinical practice, health education, and policy planning.

### Recommendations

Based on the findings of this study, several recommendations can be proposed to improve the care and support provided to menopausal women. Clinically, it is vital to incorporate routine screening for psychological distress—particularly depression and anxiety—into standard menopausal care protocols. Tools such as the DASS-21 can be utilized in gynecological and primary healthcare settings to identify at-risk women and facilitate early intervention. Multidisciplinary care models involving gynecologists, psychologists, and primary care providers are recommended to address the complex and interconnected physical and psychological needs of this population. In terms of health education, awareness campaigns and community-based programs should be developed to educate women about menopause, its potential mental health implications, and coping strategies. These initiatives can help destigmatize menopause and promote health literacy, encouraging women to seek timely medical and psychological support. Policymakers should consider developing national guidelines that integrate mental health assessments into routine menopausal care and ensure equitable access to psychological services. Future research should build on this study by employing longitudinal designs to establish causal relationships among hormonal changes, mental health, and quality of life. It is also recommended that future studies include more diverse populations and incorporate clinical assessments, such as hormone profiling, to understand the physiological underpinnings of menopausal symptoms better. A broader, integrative approach will help inform more personalized and effective care strategies for women undergoing the menopausal transition.

## Figures and Tables

**Table 1:** Distribution of study subjects according to their socio-demographic data (n = 350).

Demographic Data	No.	%
**Menopausal Status**		
Postmenopausal (≥12 months amenorrhea)	228	65.1
Perimenopausal (with symptoms but irregular menstruation)	122	34.9
**Age in years**		
45–50	73	20.9
51–55	192	54.9
56–60	85	24.3
**Mean ± SD**	**53.04 ± 7.11**	
**Level of education**		
Less than high school	50	14.3
High school degree	91	26.0
University degree	183	52.3
Post graduate	26	7.4
**Marital status**		
Single	15	4.3
Married	271	77.4
Divorced	30	8.6
Widow	34	9.7

**Table 2:** Distribution of study subjects according to their menopausal symptoms using the Menopause Rating Scale (MRS) (n = 350).

Menopause Rating Scale (MRS)	Mean	±	SD	Mild		Moderate		Severe	
				No.	%	No.	%	No.	%
**Somatic symptoms**	**3.57**	**±**	**2.26**	**123**	**35.1**	**111**	**31.7**	**116**	**32.2**
(1) Hot flashes, sweating (episodes of sweating)	1.25	±	1.06	202	57.7	105	30.0	43	12.3
(2) Heart discomfort (unusual awareness of heartbeat, skipping, heart racing, tightness)	0.90	±	0.86	261	74.6	78	22.3	11	3.1
(3) Sleep problems (difficulty in falling asleep, difficulty in sleeping through the night, waking up early)	1.42	±	1.08	175	50.0	124	35.4	51	14.6
**Psychological symptoms**	**3.55**	**±**	**2.68**	**183**	**52.3**	**113**	**32.3**	**54**	**15.4**
(4) Depressive mood (feeling down, sad, on the verge of tears, lack of drive, mood swings).	1.25	±	0.99	203	58.0	114	32.6	33	9.4
(5) Irritability (feeling nervous, inner tension, feeling aggressive)	1.16	±	1.09	222	63.4	88	25.1	40	11.4
(6) Anxiety (inner restlessness, feeling panicky)	1.15	±	1.10	233	66.6	67	19.1	50	14.3
**Urogenital symptoms**	**6.17**	**±**	**3.82**	**248**	**70.9**	**66**	**18.9**	**36**	**10.3**
(7) Physical and mental exhaustion (general decrease in performance, impaired memory, decrease in concentration, forgetfulness)	1.15	±	1.10	233	66.6	67	19.1	50	14.3
(8) Sexual problems (change in sexual desire, in sexual activity and satisfaction)	1.39	±	1.21	187	53.4	99	28.3	64	18.3
(9) Bladder problems (difficulty in urinating, increased need to urinate, bladder incontinence)	1.09	±	1.03	229	65.4	90	25.7	31	8.9
(10) Dryness of vagina (sensation of dryness or burning in the vagina, difficulty with sexual intercourse)	1.00	±	1.09	251	71.7	60	17.1	39	11.1
(11) Joint and muscular discomfort (pain in the joints. rheumatoid complaints)	1.54	±	1.09	180	51.4	102	29.1	68	19.4
**Overall**	**13.29**	±	**7.44**	99	28.3	141	40.3	110	31.4

**Table 3:** Distribution of subjects according to their quality of life during menopause (n = 350).

MENQOL Tool	Mean	±	SD	Good		Fair		Poor	
				No.	%	No.	%	No.	%
**Vasomotor symptoms**	**5.67**	**±**	**4.84**	248	70.9	76	21.7	26	7.4
1-Hot flushes or flashes	2.02	±	1.82	215	61.4	97	27.7	38	10.9
2-Night sweat	1.85	±	1.89	229	65.4	76	21.7	45	12.9
3-Sweating	1.81	±	1.73	233	66.6	91	26.0	26	7.4
**Psychosocial symptoms**	**13.58**	**±**	**9.79**	**263**	**75.1**	**69**	**19.7**	**18**	**5.1**
4-Being dissatisfied with my personal life	1.42	±	1.73	267	76.3	56	16.0	27	7.7
5-Feeling anxious or nervous	2.01	±	1.87	220	62.9	90	25.7	40	11.4
6-Experiencing poor memory	2.21	±	1.87	205	58.6	95	27.1	50	14.3
7-Accomplishing less than used to	2.42	±	1.78	191	54.6	108	30.9	51	14.6
8-Feeling depressed down or blue	1.67	±	1.76	248	70.9	72	20.6	30	8.6
9-Being impatient with other people	1.87	±	1.83	232	66.3	78	22.3	40	11.4
10-Feelings of wanting to be alone	1.97	±	1.89	221	63.1	85	24.3	44	12.6
**Physical symptoms**	**41.4**	**±**	**22.3**	**208**	**59.4**	**108**	**30.9**	**34**	**9.7**
11-Flatulence (wind)or gas pain	2.65	±	2.06	169	48.3	104	29.7	77	22.0
12-Aching in muscle and joints	3.05	±	2.06	150	42.9	92	26.3	108	30.9
13-Feeling tired or worn out	2.99	±	1.81	146	41.7	129	36.9	75	21.4
14-Difficulty sleeping	2.32	±	1.94	192	54.9	103	29.4	55	15.7
15-Aches in back of neck or head	2.54	±	2.06	184	52.6	88	25.1	78	22.3
16-Decrease in physical strength	2.99	±	1.81	142	40.6	132	37.7	76	21.7
17-Decrease in stamina	2.79	±	1.78	160	45.7	127	36.3	63	18.0
18-Feeling a lack of energy	2.92	±	1.80	149	42.6	131	37.4	70	20.0
19-Drying skin	2.82	±	1.85	169	48.3	106	30.3	75	21.4
20-Weight gain	2.71	±	2.08	179	51.1	89	25.4	82	23.4
21-Increased facial hair	1.67	±	1.89	243	69.4	67	19.1	40	11.4
22-Change in appearance, texture, or tone of your skin	2.12	±	1.92	217	62.0	83	23.7	50	14.3
23-Feeling bloated	2.48	±	2.01	185	52.9	99	28.3	66	18.9
24-Low backache	2.84	±	2.09	161	46.0	97	27.7	92	26.3
25-Frequent urination	2.35	±	1.98	199	56.9	90	25.7	61	17.4
26-Involuntary urination when laughing or coughing	2.15	±	2.16	208	59.4	72	20.6	70	20.0
**Sexual symptoms**	**6.80**	**±**	**5.75**	**220**	**62.9**	**72**	**20.6**	**58**	**16.6**
27-Change in your sexual desire	2.37	±	2.11	197	56.3	80	22.9	73	20.9
28-Vaginal dryness and during intercourse	2.10	±	2.12	217	62.0	74	21.1	59	16.9
29-Avoiding intimacy	2.33	±	2.20	199	56.9	78	22.3	73	20.9
**Overall MENQOL tool**	**67.46**	**±**	**36.85**	**252**	**72.0**	**79**	**22.6**	**19**	**5.4**

QoL categories based on sample distribution: Good ≤ 25th percentile; Fair > 25th–75th percentile; Poor > 75th percentile.

**Table 4:** Correlation between the severity of menopausal symptoms, mental health, and level of quality of life.

Variables		Overall MRS	Overall DASS21
Overall MRS	R		
	P		
Overall DASS21	R	0.346 *	
	P	<0.001 *	
Overall MENQOL	R	0.227 *	0.635 *
	P	<0.001 *	<0.001 *

*: Statistically significant at *p* ≤ 0.05.

**Table 5:** Mediating role of the Menopause Rating Scale (MRS) in the relationship between DASS-21 and Menopause-Specific Quality of Life (MENQOL) (n = 350).

Dependent Variables		Independent Variables	B	R-sq	S.E	95% Confidence Interval		t	*p* Value	Effect Proportion
						LL	UL			
Type of effect	MENQOL	MRS	0.075	0.015	0.018	−0.355	0.505	0.345	0.730	-
	MENQOL	Depression	1.535	0.526	0.046	1.162	1.908	8.139	***	34.1%
	Direct effect									
	Indirect effect		0.008	-	0.009	0.004	0.131	-	-	25.8%
	Total effect		1.543	0.127	0.041	1.174	1.913	8.700	***	-
	MENQOL	Anxiety	0.806	0.328	0.061	0.302	1.309	3.167	0.002	34.5%
	Direct effect									
	Indirect effect		0.017	-	0.054	0.018	0.874	-	-	26.0%
	Total effect		0.823	0.225	0.014	0.330	1.316	5.419	***	-
	MENQOL	Stress	1.321	0.303	0.052	0.899	1.742	6.200	***	40.5%
	Direct effect									
	Indirect effect		0.006	-	0.013	0.003	0.181	-	-	38.1%
	Total effect		1.327	0.098	0.057	0.908	1.746	5.830	***	-

B: Unstandardized regression coefficient; SE: standard error, LL: lower limit, UL: upper limit ***: statistically significant at *p* ≤ 0.001, 5000 bootstrap samples used. Effect proportion calculated as: (indirect effect/total effect) × 100. Mediation analysis performed using PROCESS macro (Hayes, 2018) [17] with 5000 bootstrap samples.

## Data Availability

The data supporting the findings of this study are available from the corresponding author upon reasonable request.
